# TLR4 dependent heparan sulphate-induced pancreatic inflammatory response is IRF3-mediated

**DOI:** 10.1186/1479-5876-9-219

**Published:** 2011-12-21

**Authors:** Hamid Akbarshahi, Jakob BF Axelsson, Katarzyna Said, Anders Malmström, Hans Fischer, Roland Andersson

**Affiliations:** 1 Department of Clinical Sciences Lund, Lund University, BMC, D12, SE-221 84 Lund, Sweden; 2 Department of Experimental Medical Sciences, Lund University, BMC, D12, SE-221 84 Lund, Sweden

**Keywords:** Heparan sulphate, pancreas, inflammation, Toll Like Receptor-4, Interferon Regulatory Factor 3

## Abstract

**Background:**

Degraded extracellular matrix can stimulate the innate immune system via the Toll-Like Receptor-4 (TLR4). In the pancreas, syndecan-anchored heparan sulphate (HS) on the ductal epithelium can be cleaved off its protein cores by the proteases (trypsin and elastase) and potentially activate TLR4 signalling.

**Methods:**

To investigate this signalling event, a low sulphated HS (500 μg/ml) was infused into the biliary-pancreatic duct of C57BL/6J wild-type mice. Phosphate buffered saline (PBS) and lipopolysaccharide (LPS) were used as negative and positive controls, respectively. Mice were sacrificed after 1, 3, 6, 9, and 48 hours and tissues were analysed for neutrophil and cytokine contents. In order to study the TLR4 signalling pathway of HS in the pancreas, genetically engineered mice lacking TLR4, Myeloid Differentiation primary response gene (88) (MyD88) or Interferon Regulatory Factor 3 (IRF3) were subjected to pancreatic infusion of HS.

**Results:**

Neutrophil sequestration and corresponding myeloperoxidase (MPO) activity in the pancreas were increased 9 hours following HS challenge. In wild-type mice, the monocyte chemoattractant protein-1(MCP-1) increased at 3 hours after infusion, while RANTES increased after 9 hours.

TLR4, MyD88, and IRF3 knockout mice showed an abrogated neutrophil recruitment and myeloperoxidase activity in the HS group, while the LPS response was only abolished in TLR4 and MyD88 knockouts.

**Conclusions:**

The results of this study show that HS is capable of initiating a TLR4-dependent innate immune response in the pancreas which is distinctly different from that induced by LPS. This inflammatory response was mediated predominantly through IRF3- dependent pathway. Release of HS into the pancreatic duct may be one important mediator in the pancreatic ductal defence.

## Background

Heparan sulphate (HS) glycosaminoglycans are complex polysaccharides which consist of a repeat disaccharide unit of uronic acid (either iduronic or glucoronic acid) linked to a glucosamine. HS is present at the cell-tissue-organ interface and has crucial regulatory roles in normal physiological processes, such as morphogenesis, tissue repair, and host defence and is usually bound covalently to different core proteins forming heparan sulphate proteoglycans (HSPGs) [1,2].

The cell surface HSPGs can act as co-receptors for soluble and insoluble ligands, soluble paracrine receptors, and internalization receptors for soluble ligands [3]. HS is found on two families of membrane-bound proteoglycans, i.e. the syndecans and glypicans. HSPGs, such as syndecan-1, are found on the epithelial cells lining the pancreatic duct [4]. Syndecans contain both HS and chondroitin sulphate chains, which vary in composition and degree of modification and differ from tissue to tissue [5].

A well-tuned defence mechanism of the pancreas is of utmost importance to the organ itself and also to the entire organism. It is possible that HSPGs are involved in the immune defence of the pancreas, but their role in pancreatitis is not well defined. However, intraductal infusion of HS induces leukocyte cell recruitment via a mechanism different from lipopolysaccharide (LPS) in a rat pancreatitis model [6]. HS can be cleaved off its protein anchors by heparinases, present in the cytosol of the pancreatic epithelial cells, and proteases (trypsin and elastase) secreted by the pancreas. Soluble HS fragments have emerged as endogenously modified and self-acting as a damage associated molecular pattern (DAMP) molecule recognized by Toll-like receptor 4 (TLR4) [7]. TLRs are also pattern recognition receptors (PRRs) and act as surveillance receptors by recognizing numerous endogenous and exogenous pathogen-associated molecular patterns (PAMPs). HS has been proposed to act as surveillance molecules, monitoring tissue integrity and function [8].

The aim of this study is to investigate the role of TLR4-signalling in HS-induced inflammatory response, as well as its downstream regulation.

## Methods

### Mice

6-8-week old male C57BL/6J, TLR4^-/- ^, MyD88^-/- ^and IRF3^-/- ^were used in the study. Mice were kept in appropriate facilities at Lund University, under specific pathogen-free conditions. The animals were kept under 12/12 hours light/dark regime in standard mesh cages and handled according to the institute guidelines with approval of the Local Animal Care Ethics Committee.

### Heparan sulphate

Heparin by-products from beef lung were treated with alkaline copper sulphate (to remove dermatan sulphate) after papain digestion. The calcium salt was then precipitated with 36% ethanol (to separate from chondroitin sulphate), followed by fractionation of the cetylpyridinium complexes by solubilisation at 0.8 M NaCl to obtain HS3. HS3 is a fraction of low sulphation (HS3, 1.00 sulphate/unit compared to 2.40 of heparin) and has lower anti-coagulant properties than heparin. In order to purify it from danger signals, such as LPS, TGF-β and other contaminating factors, the sulphated glycosaminoglycan was finally subjected to dissociative gel FPLC on Superose 6 (LKB-Pharmacia), which was eluted with 4 M guanidinium chloride 0.05 M sodium acetate, PH 5.8, at a rate of 0.4 ml/min. The effluent was analysed for glycosaminoglycans and pooled material was recovered by ethanol precipitation and converted to sodium salts [9-11].

Possible LPS contamination, which is an important consideration in the current study, was quantified by using the limulus amoebocyte lysate assay. The analysis showed a concentration of less than 0.5 EU/ml of LPS in the HS preparations. This is a concentration lower than what would be expected to influence the inflammatory response [12].

### Effects of HS in the in-vivo model

To determine the appropriate time to sample mice for the second phase of the study, a time-lapse experiment was performed with wild-type mice. The protocol for the *in vivo *experiments was a modification of a previously described procedure adopted for use in mice [13]. Briefly, the mice were anesthetized using isoflurane (Isoba Vet., Scherling-Plough, Stockholm, Sweden), a midline laparotomy was performed, the proximal end of the biliary duct clamped and the biliary-pancreatic duct was cannulated. HS 100 μl (500 μg/ml) was infused during the course of 2 minutes using an infusion pump. The clamp and the cannulae were then removed and the abdomen was closed in two layers. As a negative control, 100 μl of PBS and as a positive control, 100 μl of LPS (2.5 μg/ml from *Escherichia coli *0111:B4, Sigma, St. Louis, MO, USA) were infused. The wild-type mice (10 animals per time point) were sacrificed by exsanguination after 1, 3, 6, 9, and 48 hours, and biopsies of the duodenal lobe were harvested, snap-frozen in liquid nitrogen and stored at -70°C until further processing. For histological and immunostaining studies the samples were fixed in 4% phosphate-buffered formalin.

To investigate possible signalling pathways, experiments were performed on wild-type and 3 different strains of genetically engineered mice, TLR4^-/-^, MyD88^-/- ^and IRF3^-/-^. The *in vivo *model was performed as previously described with the exception that nine hours was used as the time point, having determined this as the peak response time from the first experiment.

The effects of a TLR4 antagonist, eritoran (E5564; Eisai Research Institute, Andover, MA), were studied. Wild-type mice were categorized in three groups (eight per group). In the first two groups, eritoran (5 mg/kg) a synthetic analogue of lipid A and an antagonist of LPS was injected into the tail vein one hour prior to laparotomy. In the last group placebo (lactose monohydrate) was injected in the same way. The first and third (placebo) group were subjected to HS3, 500 μg/ml intraductally, while the second group only underwent laparotomy, leaving the pancreas intact.

### Immunohistochemistry

Histological and immunohistochemical (IHC) techniques were followed according to standard procedures. Fixed tissue biopsies were dehydrated embedded in paraffin and 5 μm sections were cut. Primary antibodies, directed against either neutrophils (1:300, NIMP-R14, Abcam, Cambridge, UK) or F4/80 (1:500, MCA497GA, AbD Serotec, Oxford, UK) for monocytes, macrophages, and certain subpopulations of dendritic cells were used to visualize inflammatory cells. The slides were then incubated with appropriate secondary antibodies (1:400, ABC Vectastain, Vector Laboratories, Burlingame, CA, USA) and visualized using 3, 3'-diaminobenzidine (DAB, DAB peroxidase substrate Kit, Vectastain; Vector Laboratories). H_2_O_2_/methanol, levamisol (DakoCytomation) and avidin/biotin blocking (Vector Laboratories) were used to block the endogenous peroxidase, phosphatase and biotin, respectively.

Specificity of the antibodies for neutrophils and monocytes was confirmed by comparisons to morphological characters of the cell types. The slides were photographed using a Nikon Eclipse E800 microscope, Olympus DP70 camera and Nis elements software. The total number of neutrophils and monocytes in the entire sections was calculated and the total area was measured using ImageJ 1.38 (National Institute of Health, USA). Cell counts were expressed as total cells per mm^2 ^tissue.

### Myeloperoxidase activity

*Myeloperoxidase (MPO) *activity was measured according to Koike et al [14], with some modifications, as briefly outlined below. Tissue samples were homogenized and washed in gradually increasing concentrations of PBS. The supernatant was reacted with 3,3',5,5'-tetramethylbenzidine in the presence of hydrogen peroxide (H_2_O_2_) and was subsequently stopped by addition of sulphuric acid (2M, H_2_SO_4_), after which the colour shift was analysed in a spectrophotometer at 450 nm (and 540 nm as control wavelength). Horseradish peroxidase (HRP) was used as standard and the results were expressed as μU/ml.

### Cytokine production

*-Enzyme-linked immunosorbent assay (ELISA) *was used to determine concentrations of MCP-1, KC, MIP -2 and RANTES of pancreas homogenates. Homogenates were prepared by homogenizing pancreatic tissue in HEPES buffer (20 mM, pH 7.4), supplemented with EDTA (1.5 mM) and protease inhibitors (Complete, Roche Diagnostics GmbH, Mannheim, Germany). Commercially available ELISA kits were used according to the manufacturer's instructions (R&D Systems, Minneapolis, MN, USA).

### Cytokine array panel

Mouse cytokine array kit (R&D Systems, Minneapolis, USA) was used to analyse 40 different murine cytokines. Carefully selected capture antibodies have been spotted in duplicate on nitrocellulose membranes. Samples were mixed with a cocktail of biotinylated detection antibodies. The sample/antibody mixture was then incubated with the array. Chemiluminescent detection reagent was added and the signal was read.

### Statistics

The statistical analysis of the data was performed using the Mann-Whitney U-test. A p-value < 0.05 was considered statistically significant and no corrections for multiple comparisons were made. All statistical analyses were done using SPSS 16.0 (SPSS Inc., Chicago, Ill., USA). Outliers were defined as > 1.5 times the inter-quartile range and excluded from the figures, but included in all calculations.

All comparisons in the treatment groups were made to the PBS control at the corresponding time point.

## Results

### HS induces neutrophil infiltration and increased MPO activity in C57BL/6J wild-type mice

**Figure 1:**
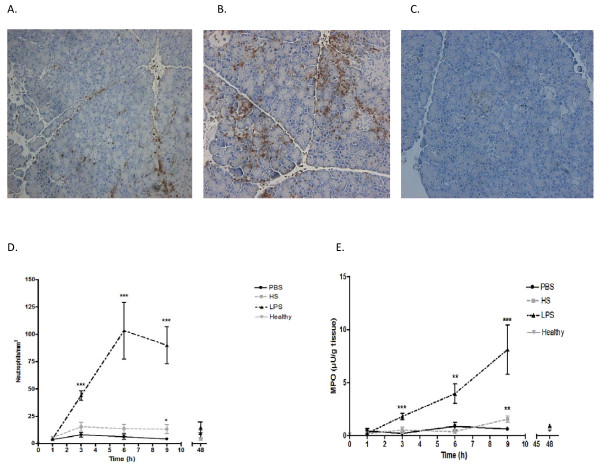
**Neutrophil infiltration into pancreatic tissue nine hours after administration of heparan sulphate (A), lipopolysaccharide (B) and PBS (C), illustrated by NIMP-R14 positive cells**. Neutrophil sequestration (D) and MPO activity (E) during 48 hours after infusion is illustrated. Neutrophil recruitment significantly increased at 3, 6 and 9 hours in the LPS treated and at 9 hours in the HS treated group compared to the PBS group. *P < 0.05 **P < 0.01 ***P < 0.001 PBS, phosphate-buffered saline, HS, heparan sulphate, LPS, lipopolysaccharide and healthy refers to untreated animals.

Immunohistochemical analysis of HS-infused pancreas showed a marked infiltration of neutrophils into the tissue after 9 hours (Figure 1A). LPS induced neutrophil recruitment into the pancreatic tissue to a higher extent (Figure 1B). No significant neutrophil staining was observed in pancreatic tissue from PBS-infused organs (Figure 1C). HS-treated organs contained a 3-fold increase in tissue neutrophil recruitment, measured as number of cells per mm^2 ^in pancreatic tissue, only 9 hours following the HS challenge as compared to the PBS group (p < 0.001; Figure 1D). The LPS-treated mice showed an increase of the neutrophil infiltration as early as at 3 hours. A further increase was noted 6 and 9 hours after the treatment (p < 0.001; Figure 1D).

Measurement of MPO activity demonstrated a similar pattern as neutrophil sequestration. MPO activity was increased at 9 hours in the HS group (mean 1.55 μU/ml, SE 0.29) as compared to the PBS group (mean 0.40 μU/ml, SE 0.17; p < 0.05). LPS caused an increase at 3, 6 and 9 hours (mean 8.11 μU/ml, SE 2.32; p < 0.001; Figure 1E).

Macrophage infiltration was also evaluated, but due to the low counts of F4/80 positive cells in each slide, no significant difference was found (data not shown).

The results showed that HS is capable of initiating an inflammatory response in the pancreatic duct, though less pronounced than seen following LPS.

### TLR4 and IRF3 are involved in HS-mediated neutrophil infiltration measured as MPO activity

**Figure 2:**
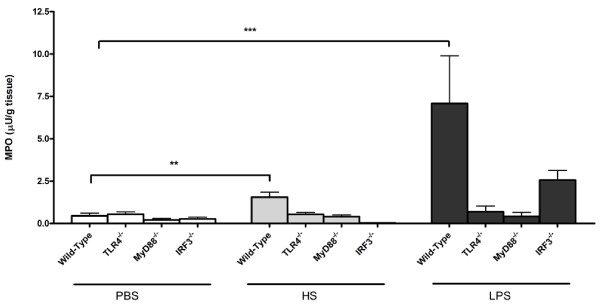
**Increased MPO activity in WT mice treated with HS**. This inflammatory response is abolished in the TLR4, MyD88 and IRF3 knockouts. *P < 0.05 **P < 0.01 ***P < 0.001.

HS as well as LPS induced significant amounts of MPO activity in the pancreatic tissue of wild-type mice (Figure 2; p < 0.05). In order to determine the role of the TLR4 signalling pathways in the HS-induced inflammatory response, TLR4^-/- ^as well as the adaptor protein, MyD88^-/- ^and the transcription factor, IRF3^-/- ^were used.

MPO activity correlated strongly with neutrophil recruitment into the tissue (Figure 1E). MPO activity was abrogated in TLR4^-/- ^mice in both HS- and LPS-treated animals, as compared to the response in the wild-type mouse. Similarly, LPS and HS did not induce an inflammatory response in MyD88^-/- ^mice (Figure 2). Interestingly, whereas the HS response in IRF3^-/- ^mice was completely abrogated, these mice still mounted a strong response to LPS (200-higher than HS-treated IRF3^-/- ^animals).

The above results show that TLR4-mediated HS inflammation is regulated by IRF3.

### Eritoran inhibits the increased MPO production in the HS-treated mouse pancreas

To investigate the mechanism by which HS activates TLR4, eritoran, a synthetic Lipid A analogue, which binds to TLR4/MD2, was used. This Lipid IVA has previously been shown to compete with Lipid A in the binding to MD2 and inhibits LPS-mediated inflammatory responses [15].

**Figure 3:**
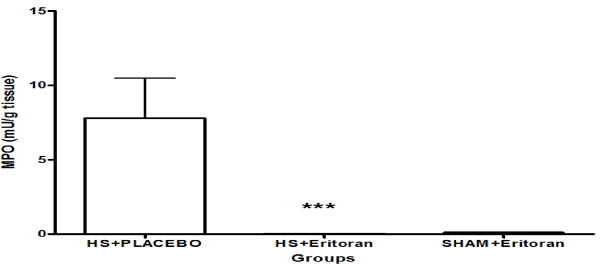
**Pre-treatment of mice with the TLR4 antagonist, eritoran significantly decreased MPO activity in HS treated mice as compared to placebo**. ***P < 0.001.

The MD2 dependence in HS-activated tissues was thus investigated using eritoran. Interestingly, the MPO activity of HS-treated wild-type mouse pancreas was completely abrogated by eritoran pretreatment (mean: 0.04177 ± 0.00405; p < 0.005) as compared to placebo (mean: 7.805 ± 2.674; Figure 3). Eritoran treatment alone did not induce any inflammatory responses.

### Chemoattractants

In search for the involved chemoattractants in the inflammatory process, pancreatic samples of the time-response study were studied for the most well-known chemoattractants of neutrophils and monocytes: the monocyte chemoattractant protein-1 (MCP-1), keratinocyte chemoattractant (KC), and macrophage inflammatory protein-2 (MIP-2).

**Figure 4:**
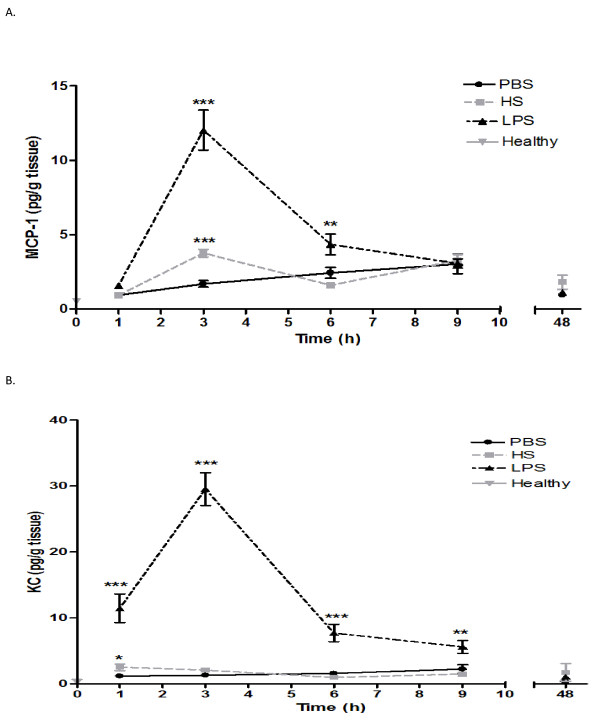
**Levels of MCP-1 (A) and KC (B) measured in HS, PBS and LPS treated mice at various time points after intraductal infusion**. *P < 0.05 **P < 0.01 ***P < 0.001.

MCP-1 significantly increased at 3 hours after infusion of HS (p < 0.001; Figure 4A). LPS induced a more pronounced response than HS, but also peaked at 3 hours after stimulation (p < 0.001; Figure 4A). The concentration thereafter decreased and reached background levels 9 hours after infusion.

The concentration of KC in the pancreatic homogenates of the HS-treated group only modestly increased after 1 hour of stimulation, but was significantly different from what was seen in animals administered PBS (p < 0.05; Figure 4B). LPS, on the other hand, induced a pronounced KC production, peaking 3 hours after stimulation (p < 0.001).

Neither HS nor LPS infusion caused any significant change in MIP-2 levels within the pancreas (data not shown).

### Cytokine array panel for IRF3^-/- ^animals

In order to provide an overview of the cytokine profile of wild-type and IRF3^-/- ^mice, we performed a cytokine array panel on the pancreas homogenates. From each group, one sample was chosen and the assay was prepared with the same amount of sample protein.

The array showed a difference between HS-treated IRF3 and WT mice regarding two cytokines: RANTES and IP10. Both cytokines were absent in the HS-treated IRF3^-/- ^mice, while present to a lesser extent in the LPS group.

**Figure 5:**
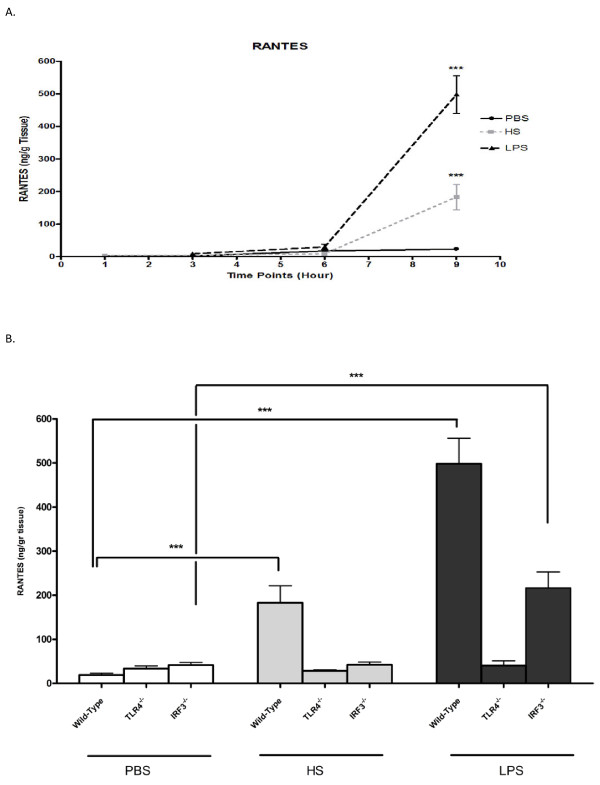
**RANTES measured at various time points in wild type mice (A)**. The level of RANTES after 9 hours of infusion of HS, PBS and LPS in wild type, TLR4 and IRF3 knockout mice (B). ***P < 0.001.

Time response samples were studied and no demonstrable differences were found in the HS-treated wild-type samples prior to nine hours (Figure 5A).

To confirm the results of the cytokine array, RANTES was measured in all samples of the wild-types, as well as in TLR4^-/- ^and IRF3^-/- ^samples. RANTES was significantly increased in the HS-treated wild-type, but not in TLR4^-/- ^and IRF3^-/- ^animals, compared to the PBS group (p < 0.001). Moreover, compared to the PBS group, the LPS-treated group had a rise in wild-type and IRF3^-/- ^mice, but not in TLR4^-/- ^mice (Figure 5B).

## Discussion

In this study we focused on the role of TLR4 and its downstream signalling in the HS-induced inflammatory response in the pancreas. We investigated the inflammatory response by HS *in vivo*. We showed that the inflammatory response in pancreas exposed to HS is TLR4-mediated. Furthermore, we extended the investigation to analyse the role of the adaptor protein MyD88 and the transcription factor IRF3 in this inflammatory response. The rationale for studying IRF3 comes from studies in the urinary tract mucosa where uropathogenic E. coli (UPEC) induces strong inflammation in a MyD88 independent manner[16]. This response to UPEC was also CD14 independent, showing activation of the mucosa by UPEC, in an LPS independent fashion [17].

Endogenous TLR activators from degraded extracellular matrix, such as biglycans, hyaluronans, HS and versicans, belong to molecules of the DAMP classification [7]. Biglycans and oligosaccharides of hyaluronan signal through TLR4 and can stimulate macrophages to produce large amounts of tumour necrosis factor-alpha (TNF-α) and MIP-2. Furthermore, antigen presenting cells such as dendritic cells (DC) were also activated by low molecular weight fragmentation products of the polysaccharides of hyaluronan through TLR4 [18,19]. Fragments of HSPG stimulate murine DC via TLR4, leading to DC maturation manifested by expression of cell surface molecules that aid in T lymphocyte stimulation [8]. This suggests that TLRs may be crucial as sensors of vital danger signals and tissue integrity [8].

To investigate if HS really acts through the TLR4 system, both an antagonist and animals specifically targeted in TLR4, or its signalling pathway, were used. Eritoran, a synthetic competitive inhibitor of LPS-induced inflammatory responses, was used. *In vitro *and *in vivo *studies have previously shown that this substance dose-dependently inhibits LPS-mediated activation of immune cells [20]. Eritoran abrogated the HS- induced inflammatory response, indicating that HS-induced dimerization of TLR4/MD2 is crucial for the inflammatory response *in vivo*. Whether HS binds directly to the receptor, acts via a co-receptor, or a mediator such as CD14 or CD 44, is not clear. This inhibitory effect may be due to binding of HS to the same position as eritoran, or a conformational change in the binding site after using eritoran, with the result that HS cannot bind and initiate signalling.

A further proof of the involvement of TLR4 and its signal transduction is the data obtained in TLR4^-/- ^animals, where the recruitment of neutrophils was abrogated. Furthermore, TLR4 involves several adaptor molecules that can lead to the initiation of inflammatory response, including the MyD88 and TRIF pathways. MyD88 is a cytosolic adaptor protein in the Toll-like receptor signalling pathway, upstream of the translocation of NF-κB into the nucleus. TRIF is the well-characterized adaptor in TLR4 signalling, which has been shown to be related to the induction of late signalling. Initiation of the TRIF-dependent pathway leads to IRF3 translocation into the nucleus, and transcription of interferon alpha and beta, as well as other interferon-induced genes [21,22].

This study is the first to show that HS-induced inflammatory response is executed through the IRF3 pathway. Neutrophil recruitment and MPO activity in the pancreas were abolished in MyD88^-/- ^and IRF3^-/- ^mice exposed to HS. However, LPS signalling was completely abrogated in MyD88^-/- ^but not in IRF3^-/- ^mice. Thus, the DAMP signal by HS promotes inflammation in the pancreatic duct differently than LPS.

In search for downstream signalling events, well-documented chemoattractants of neutrophils, KC and MIP-2, were analysed. To our surprise, no change of MIP-2, and only a slight, very early change of KC were noted after one hour. The KC change may be due to the very narrow variation rather than a relevant biological event. However MCP-1, which mainly recruits macrophages, did increase. This confirms previous reports that have suggested that leukocyte recruitment can be regulated by MCP-1 [23,24].

MCP-1 can be induced in both MyD88-dependent and -independent pathways. The IRF3 knockdown decreased the transcription of many chemokines including MCP-1 [25].

MCP-1 levels demonstrated a significant increase after three hours in the HS-treated mice. However, the macrophage counts were too low to detect significant differences between the groups.

The chemokines recruiting neutrophils activated through the IRF3 pathway are not well known. A cytokine array analysis indicated that RANTES was decreased in the IRF3^-/- ^by HS. A confirmation using ELISA showed a significant rise in the RANTES level, nine hours after the treatment with HS. This confirms earlier studies that have shown a role for RANTES in the neutrophil trafficking during inflammation [26].

In our studies HS induces a rather weak response as compared to LPS. This is what could be expected from a low-grade, but sustained response. It could be argued that this response is intended to recruit a low, but adequate number of monocytes and neutrophils to the site of cell injury. LPS, on the other hand, triggers a powerful acute response to invading pathogens where much higher amounts of inflammatory cells are recruited. As shown in the current study, LPS attracts and subsequently activates a large number of neutrophils, just as would be required in response to a bacterial infection. Our findings are in agreement with several previous reports of TLR-mediated pro-inflammatory activation cascades by DAMPs. The activation of TLR4 system by released HS leads to recruitment of leucocyte and activation of fibroblasts which is required for tissue repair. We hypothesize that, this response is a defence mechanism to remove damaged cells and further protect against activated pancreatic enzymes in the pancreatic duct.

## Conclusions

In summary, we have shown that HS is capable of initiating an innate immune response in the murine pancreas, distinct from that induced by LPS. The response is TLR4-mediated and involves both MyD88-dependent and -independent pathways. Interestingly, the HS-induced inflammation was IRF3 dependent, indicating a role of the interferon pathway in host defence response by HS. The new knowledge of an IRF3 dependent pathway by HS may prove clinically important when attempting to treat inflammations of the pancreas, with interferon signalling antagonists.

## Abbreviations

DAMP: Damage associated molecular pattern; HS: Heparan sulphate; HSPGs: Heparan sulphate proteoglycans; IRF3: Interferon Regulatory Factor 3; KC: Keratinocyte chemoattractant; LPS: Lipopolysaccharide; MCP-1- Monocyte chemotactic protein-1; MIP-2: Macrophage inflammatory protein-2; MPO: Myeloperoxidase; MyD88: Myeloid Differentiation primary response gene (88); PAMPs: Pathogen associated molecular patterns; PBS: Phosphate-buffered saline; PRR: Pattern recognition receptor; TLR-4: Toll like receptor-4; UPEC: Uropathogenic E.coli.

## Competing interests

The authors declare that they have no competing interests.

## Authors' contributions

HA, JA, KS were involved in the design of the experiment, carrying out the experimental work and writing the manuscript. AM, HF and RA were involved in the design the study as well as funding it and writing the manuscript.

All coauthors have read and approved the final manuscript.
